# Influence of the COVID-19 pandemic on pain and oral health-related quality of life in women with temporomandibular disorder

**DOI:** 10.1590/2177-6709.27.3.e2220422.oar

**Published:** 2022-07-04

**Authors:** Ana Karolina Reis MENDONÇA, Luana Pinheiro Guerra FONTOURA, Thaynara Domingos da ROCHA, Rocharles Cavalcante FONTENELE, Tereza Nicolle Burgos NUNES, Romulo Rocha REGIS, Lívia Maria Sales PINTO-FIAMENGUI

**Affiliations:** 1 Universidade Federal do Ceará, Faculdade de Odontologia (Fortaleza/CE, Brazil).; 2 Universidade Estadual de Campinas (Campinas/SP, Brazil).; 3 Universidade Federal do Ceará, Departamento de Odontologia Restauradora (Fortaleza/CE, Brazil).

**Keywords:** Coronavirus, Quality of life, Facial pain

## Abstract

**Objective:**

This study aimed to evaluate and compare pain intensity and Oral Health-related Quality of Life (OHRQoL) in women with temporomandibular disorder (TMD) before (T1) and during (T2) COVID-19 pandemic.

**Materials and Methods:**

Sample was composed of forty-one female participants with painful TMD, who presented for TMD treatment. Subjects were asked to indicate their pain intensity and to answer the Oral Health Impact Profile-14 (OHIP-14). Participants data were collected twice: T1 (evaluation of medical records fulfilled before COVID-19 pandemic) and T2 (by means of an online form). Socio-demographic data were assessed in T1. Statistical analysis was performed with a significance level of 5% (Wilcoxon, chi-square or Fisher’s exact tests, multiple linear regressions).

**Results:**

No difference was found in pain intensity (*p*=0.26) and OHIP-14 global scores (*p*=0.53). Physical pain (*p*=0.03) and social disability (*p*=0.05) domains improved. In T1, subject’s occupation was associated with OHIP-14 global score, physical pain, and physical disability domains. In T2, age was associated with OHIP-14 global scores as well as physical pain, psychological discomfort, and psychological disability domains.

**Conclusion:**

COVID-19 pandemic did not worsen pain intensity and OHRQoL in women with painful TMD, and it is suggested that socio-demographic characteristics influenced TMD patients coping skills during pandemic.

## RESUMO

**Objetivo:**

O presente estudo objetivou avaliar e comparar a intensidade da dor e a Qualidade de Vida Relacionada à Saúde Bucal (QVRSB) de mulheres com disfunção temporomandibular (DTM), antes (T1) e durante (T2) a pandemia de COVID-19.

**Métodos:**

A amostra foi composta por quarenta e uma mulheres com DTM dolorosa, que se apresentaram para manejo da DTM. As participantes informaram a intensidade da dor por meio de escala numérica, e responderam ao questionário Perfil de Impacto na Saúde Oral - 14 (*Oral Health Impact Profile*, OHIP-14). Os dados das participantes foram coletados duas vezes: T1 (avaliação de prontuários odontológicos preenchidos previamente à pandemia de COVID-19) e T2 (por meio de formulário on-line). Dados sociodemográficos foram coletados em T1. A análise estatística foi realizada com nível de significância de 5% (testes de Wilcoxon, qui-quadrado ou exato de Fisher, e regressões lineares múltiplas).

**Resultados:**

Nenhuma diferença foi encontrada para a intensidade da dor (*p* = 0,26) e o escore global do OHIP-14 (*p* = 0,53). Os domínios relativos à dor física (*p* = 0,03) e incapacidade social (*p* = 0,05) melhoraram. Em T1, a ocupação das participantes foi associada ao escore global do OHIP-14 e aos domínios de dor e incapacidade física. Em T2, a idade foi associada aos escores globais do OHIP-14, bem como aos domínios dor física, desconforto psicológico e incapacidade psicológica.

**Conclusão:**

A pandemia de COVID-19 não piorou a sensibilidade à dor e a QVRSB das mulheres com DTM dolorosa, e as características sociodemográficas influenciaram suas habilidades de enfrentamento.

## INTRODUCTION

On 31 December 2019, the World Health Organization China Country Office^1^ was informed of cases of pneumonia of unknown etiology detected in Wuhan, Hubei, China. Later, the disease was confirmed to be caused by a novel coronavirus (SARS-CoV-2) and was designated as coronavirus disease 2019 (COVID-19). On March 11, 2020, it reached the level of a pandemic, affecting countries across the world.¹ Due to its high contagion potential,^2^ public health measures such as social isolation and quarantine were implemented to minimize the virus transmission.^3^

While the scientific community is focusing mainly on COVID-19 prevention and treatment,^2^ ^,^ ^4^ several psychological and emotional issues are being underestimated^1^ ^,^ ^4^ and those may last longer than the disease itself.^2^ Individual and community psychosocial impacts associated with a pandemic include fear,^5^ ^,^ ^6^ ^,^ ^7^ anxiety,^4^ ^-^ ^11^ depression,^5^ ^,^ ^8^ ^-^ ^11^ panic,^6^ ^,^ ^8^ ^,^ ^11^ stress,^5^ ^,^ ^6^ ^,^ ^9^ ^-^ ^11^ and episodes of post-traumatic stress.^7^ ^,^ ^12^ However, up to the date of this study, studies investigating the impact of the pandemic on quality of life are lacking.^13^

Psychological factors are directly related to chronic painful conditions, and the COVID-19 pandemic has many characteristics that could potentially worsen these conditions.^5^ Temporomandibular disorder (TMD) is a common musculoskeletal disorder resulting in pain and disability, and causes a significant impact on the individual as well as society, due to loss of productivity and increased number of consultations required for diagnosis and patient care.^14^

TMD patients suffer from various types of psychosocial distress, such as anxiety, depression, and suicidal ideation.^15^ Particularly in times of crisis, such as the COVID-19 pandemic, special attention must be given to vulnerable patient groups - not only those susceptible to SARS-CoV-2, but also to those mentally unstable.

Individuals with TMD also present impaired oral health-related quality of life (OHRQoL).^16^ ^-^ ^19^ OHRQoL is a conceptual model targeting the individual’s perception of oral health.^18^ It brings together the multidimensional character of symptoms, perceptions, and functional capacity.^16^ The Oral Health Impact Profile (OHIP) is the most widely used instrument for measuring OHRQoL,^16^ and when compared to healthy individuals, patients with TMD present worse scores related to physical pain, discomfort, and psychological disabilities.^18^

Mental health can be defined by how individuals think and feel about themselves and their life, and it affects how an individual copes and manages in times of adversity. Physical and mental health are closely related, as they affect each other directly or indirectly.^20^ Most mental health studies on COVID-19 focus on general population;^4^ ^,^ ^6^ ^,^ ^8^ ^-^ ^12^ ^,^ ^13^ however, psychological and behavioral changes due to the pandemic reveals the need for studies within specific populations, such as those with painful conditions. Therefore, due to the already established association between the COVID-19 pandemic and psychosocial impairment,^2^ ^,^ ^4^ ^,^ ^5^ ^,^ ^10^ ^,^ ^12^ and the role of these in TMD worsening and perpetuation,^14^ ^-^ ^18^ this longitudinal study aimed to evaluate and compare pain intensity and OHRQoL of women with TMD before and during COVID-19 pandemic.

## METHODS

This was a cross-sectional study approved by Ethics Committee of Universidade Federal do Ceará (CAAE 11863019.6.0000.5054). Subjects were recruited among those seeking for TMD treatment at Federal University of Ceará, in 2019. Eligible participants were women living in Fortaleza (Ceará, Brazil) during pre-pandemic and pandemic periods, aged between 18 and 55 years old, alphabetized, presenting Internet access, and diagnosed with at least one painful TMD according to the Research Diagnostic Criteria for Temporomandibular Disorders (RDC/TMD). Exclusion criteria were presence of other chronic painful disorders, licit or illicit drug abuse, alcohol abuse, and cognitive impairment. All participants read and signed the informed consent form before entering the study.

### OUTCOME VARIABLES

Participants data were collected twice: T1 (through evaluation of medical records fulfilled before COVID-19 pandemic, from August 1st to November 30, 2019) and T2 (by means of an online form, Google Form Survey, sent by e-mail and fulfilled during COVID-19 pandemic, from April 24 to May 1st, 2020). Subjects were asked to indicate their pain intensity in an eleven-point Numeric Scale (NS) ranging from “0” (“no pain”) to 10 (“worst pain imaginable”), and to answer the Portuguese version of OHIP-14 form.^21^ OHIP-14 consists of 14 items including seven conceptual domains (2 items each) of OHRQoL: functional limitation, physical pain, psychological discomfort, physical disability, psychological disability, social disability, and handicap. For each item, subjects were asked to answer how frequently they had adverse impacts caused by TMD during the previous month, according to a 5-point ordinal scale, being: 0 = never, 1 = hardly ever, 2 = occasionally, 3 = fairly often, and 4 = very often. The obtained scores were used to calculate severity of OHRQoL impairment.^19^ Severity was characterized by the OHIP-14 global score, with a potential range of 0 (no adverse impacts) to 56 (all 14 impacts experienced very often). OHIP-14 domains were calculated by summing the response scores for the two corresponding items.^18^ Socio-demographic characteristics were assessed in T1 (age, ethnicity, marital status, educational level, family income, and occupation) and T2 (occupation during pandemic).

### POWER ANALYSIS OF THE SAMPLE

As this study used a convenience sample, the *post-hoc* power analysis was performed using the G Power software v. 3.1.9.2 (Faz Faul, Kiel University, Germany). Considering the difference of the mean and the standard deviation calculated between T1 and T2 for all parameters evaluated and a significance level (α) of 0.05, it was obtained a test’s power of 0.84.

### STATISTICAL ANALYSIS

Data were collected by a single operator and presented as mean ± standard deviation (SD) for the quantitative variables, and percentage for the qualitative variables. The normality of the data was assessed using the Kolmogorov-Smirnov test. Wilcoxon test was applied to compare OHIP-14 global score, OHIP-14 individual domains and the NS scores obtained in T1 and T2. Socio-demographic and clinical factors were assessed by the Chi-square or Fisher’s exact tests. Multiple linear regressions analyses were performed to clarify the association of socio-demographic and clinical factors on OHIP-14 severity before and during pandemic. All analyses were performed using SPSS software v. 24.0 (IBM, Corp., Armonk, NY) with a α=0.05.

## RESULTS

### GENERAL DESCRIPTION

294 female volunteers were evaluated, from which 61 subjects were confirmed for eligibility and included in the study. Data from all subjects were collected in T1, and 41 completed the follow-up (T2) (67.2% response rate of online form). From those who did not complete the study, reasons were as follows: loss of contact = 7; refused to participate = 8; agreed to participate, but did not answer the questionnaire = 6. Participants mean age (T1) was 26.83 ± 7.54 years. Most participants were aged less than 30 years, were white, single, with a high school educational formation, and no occupation during pandemic. A detailed description of socio-demographic characteristics is presented in Table 1. 

**Table 1:** Sample characterization.

Variables	n (%)	p-value
Age^a^		
< 30-years	27 (61.8)	0.04*
≥ 30-years	14 (34.2)	
Ethnicity /skin color^b^		
White	23 (56.1)	< 0.001*
Black	1 (2.4)	
Yellow	3 (7.3)	
Brown	14 (34.2)	
Marital status^b^		
Single	36 (87.8)	< 0.001*
Divorced	1 (2.4)	
Married	4 (9.8)	
Education level^b^		
Less than high school	3 (7.3)	< 0.001*
High school	26 (63.4)	
Graduate or higher	12 (29.3)	
Family income (minimum wage)^b^		
< 1	6 (14.6)	0.31
1-1.9	5 (12.2)	
2-2.9	7 (17.1)	
3-5	11 (26.8)	
> 6	12 (29.3)	
Occupation^a^		
Graduate student	17 (41.5)	0.08
Health professional	7 (17)	
Other professions	17 (41.5)	
Occupation during pandemic^a^		
Unaltered	10 (24.4)	0.001*
Home-office	6 (14.6)	
No occupation	25 (61.0)	
TMD treatment after first OHRQoL evaluation^a^		
Yes	19 (46.3)	0.64
No	22 (53.7)	

Brazilian minimum wage in 2020: R$1045.00

*Significant difference (p ≤ 0.05).

^a^ Chi-square or ^b^ Fisher’s exact test.

**Figure 1:**
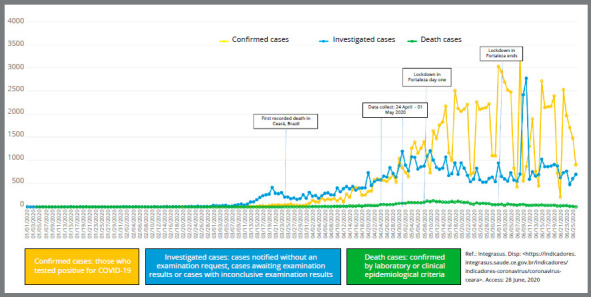
Regional trend of COVID-19 pandemic in Ceará, Brazil, from January 1 to June 25, 2020.

The regional evolution of COVID-19 pandemic from January 1^st^ to June 26^th^, 2020, as well as the period of subject’s second evaluation (T2) are shown in Figure 1.

### OUTCOME VARIABLES

According to the NS, there was no difference in pain intensity between evaluations (T1=5.95 ± 1.78; T2=5.42 ± 2.62; *p*=0.26). The OHIP-14 global and domains scores are presented in Table 2. No difference in OHIP-14 global score was found (*p*=0.53), however, scores related to physical pain (*p*=0.03) and social disability (0.05) decreased, and scores related to functional limitation increased (*p*=0.02).

**Table 2:** Mean Oral Health Impact Profile (OHIP-14) global and domain scores before (T1) and during (T2) COVID-19 pandemic.

OHIP-14 Score	T1 (n = 41)	T2 (n = 41)	p-value
	Mean (SD)	Mean (SD)	
Global score	22.27 (8.91)	21.07 (12.33)	0.53
Functional limitation*	0.71 (0.96)	1.37 (1.77)	0.02
Physical pain*	5.17 (1.50)	4.22 (2.24)	0.03
Psychological discomfort	4.8 (1.96)	4.83 (2.34)	0.64
Physical disability	2.76 (2.08)	2.78 (2.25)	0.96
Psychological disability	3.34 (1.62)	3.32 (2.11)	0.99
Social disability*	3.32 (2.36)	2.51 (1.87)	0.05
Handicap	2.00 (2.17)	2.10 (1.85)	0.94

* Significant difference p ≤ 0.05; Wilcoxon test.

Tables 3 and 4 show the results regarding the multiple linear regressions for the periods before (T1) and during (T2) pandemic, respectively. In T1, subject’s occupation was associated with OHIP-14 global score, physical pain, and physical disability domains (*p*<0.05). Nevertheless, the combination of family income and marital status showed a significant association with handicap domain (*p*<0.05).

**Table 3:** Multiple linear regression of Oral Health Impact Profile (OHIP-14) scores before COVID-19 pandemic (T1) according to socio-demographic characteristics.

OHIP-14 scores (T1)						
Socio-demographic factors	B (SE)	CI (95%)	R	R^2^	F	p-valor
Professional activity	*Global score*					
	3.41 (1.45)	(0.48 - 6.34)	0.35	0.12	5.55	0.02
	*Physical pain*					
	0.88 (0.21)	(0.44 - 1.32)	0.54	0.29	16.3	<0.001
	*Physical disability*					
	0.88 (0.33)	(0.21 - 1.56)	0.39	0.15	7.02	0.01
	*Handicap*					
Family income	-0.48 (0.20)	(-0.90 - 0.69)	0.57	0.32	5.58	0.001
Marital status	1.70 (0.47)	(0.74 - 2.65)				0.02

SE = standard error; CI = confidence interval; R² = coefficient of determination.

The independent variables did not show a significant (p ≥ 0.05) association with the other domains (Functional limitation, Psychological discomfort, Psychological disability, and Social disability) of OHIP-14.

**Table 4:** Multiple linear regression of Oral Health Impact Profile (OHIP-14) scores during COVID-19 pandemic (T2), according to socio-demographic characteristics.

Escores OHIP-14 (T2)						
Sociodemographic factors	B (SE)	CI (95%)	R	R^2^	F	p-value
Age	*Global score*					
	10.52 (3.75)	(2.92 - 18.11)	0.41	0.17	7.85	0.008
	*Physical pain*					
	2.27 (0.65)	(0.95 - 3.60)	0.49	0.24	12.07	0.001
	*Psychological discomfort*					
	2.21 (0.70)	(0.80 - 3.62)	0.45	0.2	10.07	0.003
	*Social disability*					
	1.28 (0.59)	(0.09 - 2.47)	0.33	0.11	4.73	0.04
	*Psychological disability*					
	1.90 (0.64)	(0.62 - 3.19)	0.43	0.19	8.97	0.005
Marital status	*Functional limitation*					
	1.04 (0.43)	(0.17 - 1.92)	0.36	0.13	5.87	0.02

SE = standard error; CI = confidence interval; R² = coefficient of determination.

The independent variables did not show a significant (p ≥ 0.05) association with the other domains (Physical disability and Handicap) of OHIP-14.

In T2, subject’s age was associated with OHIP-14 global scores, as well as with the individual domains physical pain, psychological discomfort, social disability, and psychological disability (*p*<0.05). Subjects over 30 years of age presented worse OHRQoL (R values ranged from 0.33 to 0.49). Furthermore, marital status was associated with functional limitation (*p*<0.05). 

## DISCUSSION

This study aimed to evaluate and compare pain intensity and OHRQoL of women with painful TMD before and during COVID-19 pandemic. The influence of socio-demographic factors in OHRQoL was also assessed. Furthermore, unlike several studies evaluating psychological impact of COVID-19 pandemic,^4^ ^,^ ^6^ ^,^ ^8^ ^,^ ^11^ ^,^ ^12^ the present study reported a pre-pandemic baseline data. No difference was found in pain intensity and OHIP-14 global scores, and physical pain and social disability individual domains improved. Before and during pandemic, OHIP-14 global scores and individual domains were distinctly associated with socio-demographic characteristics. Before pandemic, subject’s occupation was associated with OHIP-14 global score, physical pain, and physical disability domains. The handicap domain was associated with a combination of both family income and marital status. On the other hand, during pandemic, age was associated with OHIP-14 global scores as well as with individual domains: physical pain, psychological discomfort, social disability and psychological disability. Lastly, marital status influenced functional limitation domain. 

According to Almeida-Leite et al,^22^ it could be expected that psychological factors associated to the COVID-19 pandemic would lead to greater risk of developing, worsening, and perpetuating TMD, especially due to the already established influence of those factors on patients pain and somatization.^23^ Reduced accessibility to regular medical care during the social isolation due to pandemic should also be pointed out as a risk factor for the worsening of painful disorders.^5^ In the present study, however, conflicting results were found, since subject’s pain intensity and OHRQoL were similar before and during pandemic, and scores of OHIP-14 physical pain domain improved. Findings presented here suggest substantial need to consider socio-demographic characteristics when dealing with TMD patients.

In the present study, all participants were engaged in a regular occupational activity before the COVID-19 pandemic. Work-related factors, such as occupation^24^ ^,^ ^25^ and working-hours^26^ have been related to TMD. Professions such as computer office workers^24^ ^,^ ^25^ and dentists^25^ show increased risk of developing TMD. In a previous study, the risk of TMD was found to be higher among women working more than 60h per week than among those working less than 40h per week, after adjusting for the general characteristics and work-related factors.^26^ Also, TMD prevalence rate was higher for workers who perceived stress a lot.^26^ Long-working hours have also been related to anxiety and depression,^27^ and it may be assumed that mental and physical stress arising from long working-hours may affect TMD patients.^26^ During pandemic, most subjects (61%) were presenting no occupation. A nationwide survey of psychological distress among Italian people during the COVID-19 pandemic found, among other factors, higher levels of stress were associated with having to leave one’s domicile for work.^9^ Therefore, staying in a safe place, as home, may have been a protective factor for most subjects of the present study, since perceived stress is also a risk factor for TMD.^28^ Perhaps, the reduction of occupational activity, and reduced need of being productive in the work place, may have improved physical pain and social disability domains, and prevented subjects from TMD worsening.

According to a systematic review, the most often-affected OHIP domains in TMD patients are those evaluating psychological discomfort and disability, while social disability and handicap are the least often affected.^16^ Although social isolation and quarantine were considered to cause a negative impact on many aspects of people’s lives,^9^ in the present study, the social disability domain improved during pandemic. A previous study conducted among the general population living in Jinzhou, Lianing Province, China, found that during pandemic, the majority of participants reported receiving increased social support from family and friends and increased caring for family members, especially those aged between 18-40 years.^13^ In the present study those variables were note analyzed, however, the quarantine and the pandemic itself may have given opportunity for people to support and care for each other.

In the present study, participants were categorized into two age groups, and the cut-off point was set at 30 years.^17^ During pandemic, age was associated to OHIP-14 global scores as well as for physical pain, psychological discomfort, and psychological disability individual domains. According to the literature, young adults (aged 18-30 years) and older adults (over 60 years) exhibited highest levels of psychological distress during pandemic.^11^ It was suggested that greater psychological distress in younger population might have occurred due to greater access to information through social media, which can easily trigger stress.^9^ Here, the presence of comorbidities was not assessed, but individuals presenting 30-years or more includes those that are more likely to present comorbidities that increases COVID-19 patient’s risk, such as hypertension, diabetes, chronic obstructive pulmonary disease, and cerebrovascular disease.^29^ Also, in Brazil, the most affected age group was 30-39 years,^30^ therefore, the fear of infection may have impacted OHRQoL related to TMD of subjects over 30 years-old. 

This study presents some limitations, such as small sample size and the convenience sampling method. Additionally, online form application is restricted to those with Internet access, and selection bias may have occurred. Possibly, answering the online form during pandemic was restricted to those who suffered modest socioeconomic and emotional impact. Also, although socioeconomic and demographic characteristics were collected at baseline, only information regarding changes in occupation was collected during pandemic, which may have influenced the findings. Therefore, results presented here should not be generalized. Variables, such as if subjects were quarantined with family or alone, use of social media, amount of health information, subjects who were infected by COVID-19, deaths among loved ones, history of medical problems^9^ were not assessed. In addition, variables known to influence TMD pain, such as physical activity and sleep quality were not evaluated. 

## CONCLUSION

COVID-19 pandemic did not worsen pain intensity and OHRQoL in women with painful TMD, and it is suggested that socio-demographic characteristics influenced TMD patients coping skills during pandemic. More studies in the context of a biopsychosocial model during and after the pandemic are needed to better elucidate its impact on TMD patients pain severity and OHRQoL, and also to point out some learning for future global outbreaks or even future waves of COVID-19.
